# 3-{2-[(3-{(*E*)-2-[4-(Dimethyl­amino)­phen­yl]ethen­yl}quinoxalin-2-yl)­oxy]eth­yl}-1,3-oxazolidin-2-one monohydrate

**DOI:** 10.1107/S1600536811054481

**Published:** 2011-12-23

**Authors:** Youssef Ramli, Hafid Zouihri, El Mokhtar Essassi, Seik Weng Ng

**Affiliations:** a Laboratoire de Chimie Organique Hétérocyclique, Pôle de Compétences Pharmacochimie, Université Mohammed V-Agdal, BP 1014 Avenue Ibn Batout, Rabat, Morocco; b CNRST Division UATRS, Angle Allal Fassi/FAR, BP 8027 Hay Riad, Rabat, Morocco; c Department of Chemistry, University of Malaya, 50603 Kuala Lumpur, Malaysia; d Chemistry Department, King Abdulaziz University, PO Box 80203 Jeddah, Saudi Arabia

## Abstract

In the title compound, C_23_H_24_N_4_O_3_·H_2_O, the 1,3-oxazoline ring is nearly planar [maximum deviation = 0.059 (2) Å] and its mean plane is twisted by 30.12 (8)° with respect to the quinoxaline fused-ring system; the benzene ring is nearly coplanar with the quinoxaline fused-ring system [dihedral angle = 2.52 (2)°]. The water mol­ecule of crystallization is hydrogen-bond donor to an N atom of the quinoxaline ring system as well as an O atom of the oxazolinone unit, the two hydrogen bonds generating a chain running along the *c* axis.

## Related literature

For general background, see: Noolvi *et al.* (2011 ▶).
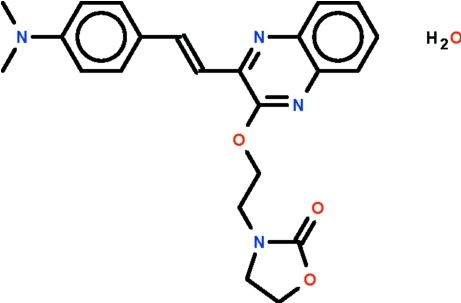


## Experimental

### 

#### Crystal data

C_23_H_24_N_4_O_3_·H_2_O
*M* *_r_* = 422.48
Monoclinic, 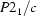

*a* = 7.20980 (1) Å
*b* = 23.3271 (4) Å
*c* = 12.3994 (2) Å
β = 98.119 (1)°
*V* = 2064.47 (6) Å^3^
*Z* = 4
Mo *K*α radiation
μ = 0.10 mm^−1^
*T* = 293 K
0.21 × 0.20 × 0.15 mm

#### Data collection

Bruker APEX DUO diffractometer
28134 measured reflections
5836 independent reflections
4141 reflections with *I* > 2σ(*I*)
*R*_int_ = 0.031

#### Refinement

*R*[*F* ^2^ > 2σ(*F* ^2^)] = 0.046
*wR*(*F* ^2^) = 0.137
*S* = 1.03
5836 reflections
290 parameters
2 restraints
H atoms treated by a mixture of independent and constrained refinement
Δρ_max_ = 0.48 e Å^−3^
Δρ_min_ = −0.25 e Å^−3^

### 

Data collection: *APEX2* (Bruker, 2010 ▶); cell refinement: *SAINT* (Bruker, 2010 ▶); data reduction: *SAINT*; program(s) used to solve structure: *SHELXS97* (Sheldrick, 2008 ▶); program(s) used to refine structure: *SHELXL97* (Sheldrick, 2008 ▶); molecular graphics: *X-SEED* (Barbour, 2001 ▶); software used to prepare material for publication: *publCIF* (Westrip, 2010 ▶).

## Supplementary Material

Crystal structure: contains datablock(s) global, I. DOI: 10.1107/S1600536811054481/xu5414sup1.cif

Structure factors: contains datablock(s) I. DOI: 10.1107/S1600536811054481/xu5414Isup2.hkl

Supplementary material file. DOI: 10.1107/S1600536811054481/xu5414Isup3.cml

Additional supplementary materials:  crystallographic information; 3D view; checkCIF report

## Figures and Tables

**Table 1:** Hydrogen-bond geometry (Å, °)

*D*—H⋯*A*	*D*—H	H⋯*A*	*D*⋯*A*	*D*—H⋯*A*
O1w—H11⋯N1	0.87 (1)	2.18 (1)	3.039 (2)	170 (2)
O1w—H12⋯O3^i^	0.86 (1)	1.96 (1)	2.820 (2)	176 (3)

Symmetry code: (i) 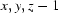.

**Fig. 1.**
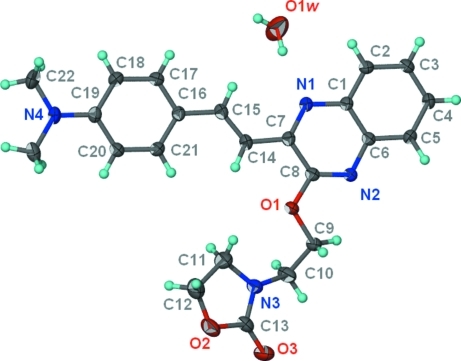
Thermal ellipsoid plot (Barbour, 2001) of C23H24N4O3.H2O at the 70% probability level; hydrogen atoms are drawn as spheres of arbitrary radius.

## supplementary crystallographic information

### Comment

2-Chloro-3-(4-dimethylaminostyryl)quinoxaline is a reactant for the synthesis of quinoxaline derivatives that possess anti-cancer properties (Noolvi *et al.*, 2011); the chloro substitutent is exchanged for other organic radicals. We have used the 3-(4-dimethylaminostyryl)quinoxaline as reactant to furnish the title derivative (Scheme I). The quinoxaline fused-ring and the phenylene ring are nearly co-planar (dihedral angle 2.52 (2)°) (Fig. 1). The water molecule is hydrogen bond donor to an N atom of the fused-ring as well as an O atom of the oxazolinone unit, the two hydrogen bonds generating a linear chain running along the *c*-axis of the monoclinic unit cell (Table 1).

### Experimental

To 3-(4-dimethylaminostyryl)quinoxaline (1 g, 3.43 mmol), potassium carbonate (0.71 g, 5.15 mmol) and a catalytic amount of tetra-*n*-butylammonium bromide in DMF (40 ml) was added bis(2-chloroethyl)amine hydrochloride (1.22 g, 6.87 mmol). The mixture was heated for 48 hours. After the completion of the reaction (as monitored by TLC), the inorganic material salt was filtered and the solvent was removed under reduced pressure. The solid product was purified by recrystallization from ethanol to afford colorless crystals.

### Refinement

Carbon-bound H-atoms were placed in calculated positions (C–H 0.93–0.97 Å) and were included in the refinement in the riding model approximation, with *U*(H) set to 1.2–1.5*U*(C).

The water H-atoms were located in a difference Fourier map and were refined with a distance restraint of O–H 0.84±0.01 Å; their temperature factors were refined.

Omitted were 0 1 1 and 0 2 0.

### Crystal data

**Table:**

C_23_H_24_N_4_O_3_·H_2_O	*F*(000) = 896
*M**_r_* = 422.48	*D*_x_ = 1.359 Mg m^−^^3^
Monoclinic, *P*2_1_/*c*	Mo *K*α radiation, λ = 0.71073 Å
Hall symbol: -P 2ybc	Cell parameters from 7579 reflections
*a* = 7.20980 (1) Å	θ = 3.0–30.1°
*b* = 23.3271 (4) Å	µ = 0.10 mm^−^^1^
*c* = 12.3994 (2) Å	*T* = 293 K
β = 98.119 (1)°	Prism, colorless
*V* = 2064.47 (6) Å^3^	0.21 × 0.20 × 0.15 mm
*Z* = 4	

### Data collection

**Table:**

Bruker APEX DUO diffractometer	4141 reflections with *I* > 2σ(*I*)
Radiation source: fine-focus sealed tube	*R*_int_ = 0.031
graphite	θ_max_ = 30.1°, θ_min_ = 2.4°
ω scans	*h* = −10→10
28134 measured reflections	*k* = −32→27
5836 independent reflections	*l* = −17→17

### Refinement

**Table:**

Refinement on *F*^2^	Primary atom site location: structure-invariant direct methods
Least-squares matrix: full	Secondary atom site location: difference Fourier map
*R*[*F*^2^ > 2σ(*F*^2^)] = 0.046	Hydrogen site location: inferred from neighbouring sites
*wR*(*F*^2^) = 0.137	H atoms treated by a mixture of independent and constrained refinement
*S* = 1.03	*w* = 1/[σ^2^(*F*_o_^2^) + (0.0694*P*)^2^ + 0.5463*P*] where *P* = (*F*_o_^2^ + 2*F*_c_^2^)/3
5836 reflections	(Δ/σ)_max_ = 0.001
290 parameters	Δρ_max_ = 0.48 e Å^−^^3^
2 restraints	Δρ_min_ = −0.25 e Å^−^^3^

### Special details

**Table:**

Geometry. All esds (except the esd in the dihedral angle between two l.s. planes) are estimated using the full covariance matrix. The cell esds are taken into account individually in the estimation of esds in distances, angles and torsion angles; correlations between esds in cell parameters are only used when they are defined by crystal symmetry. An approximate (isotropic) treatment of cell esds is used for estimating esds involving l.s. planes.

### Fractional atomic coordinates and isotropic or equivalent isotropic displacement parameters (Å^2^)

**Table:**

	*x*	*y*	*z*	*U*_iso_*/*U*_eq_	
O1	0.69604 (14)	0.57099 (4)	0.70080 (7)	0.0248 (2)	
O2	0.68168 (18)	0.42279 (4)	0.99270 (9)	0.0414 (3)	
O3	0.74191 (19)	0.50321 (5)	1.08888 (9)	0.0436 (3)	
O1W	0.8486 (2)	0.59976 (6)	0.21995 (13)	0.0614 (4)	
H11	0.797 (3)	0.5986 (11)	0.2789 (14)	0.080 (8)*	
H12	0.813 (4)	0.5698 (8)	0.1822 (19)	0.088 (9)*	
N1	0.65125 (15)	0.61020 (4)	0.42003 (9)	0.0201 (2)	
N2	0.60511 (15)	0.65809 (4)	0.62458 (8)	0.0200 (2)	
N3	0.73026 (18)	0.50212 (5)	0.90253 (9)	0.0271 (3)	
N4	0.95701 (19)	0.25635 (5)	0.38066 (10)	0.0296 (3)	
C1	0.59653 (17)	0.66630 (5)	0.42868 (10)	0.0179 (2)	
C2	0.56427 (19)	0.70093 (5)	0.33486 (10)	0.0226 (3)	
H2	0.5819	0.6858	0.2677	0.027*	
C3	0.5071 (2)	0.75683 (5)	0.34146 (10)	0.0238 (3)	
H3	0.4860	0.7793	0.2790	0.029*	
C4	0.48053 (19)	0.78010 (5)	0.44295 (11)	0.0229 (3)	
H4	0.4410	0.8179	0.4471	0.027*	
C5	0.51243 (19)	0.74744 (5)	0.53594 (10)	0.0217 (3)	
H5	0.4945	0.7632	0.6026	0.026*	
C6	0.57205 (17)	0.69021 (5)	0.53078 (10)	0.0178 (2)	
C7	0.68059 (18)	0.57926 (5)	0.50954 (10)	0.0189 (2)	
C8	0.65714 (18)	0.60568 (5)	0.61326 (10)	0.0194 (3)	
C9	0.6853 (2)	0.59576 (5)	0.80636 (10)	0.0265 (3)	
H9A	0.5564	0.5963	0.8205	0.032*	
H9B	0.7320	0.6348	0.8091	0.032*	
C10	0.8030 (2)	0.55941 (6)	0.88980 (11)	0.0287 (3)	
H10A	0.9277	0.5562	0.8695	0.034*	
H10B	0.8145	0.5788	0.9596	0.034*	
C11	0.7117 (3)	0.45883 (6)	0.81770 (12)	0.0360 (4)	
H11A	0.8293	0.4526	0.7899	0.043*	
H11B	0.6155	0.4690	0.7579	0.043*	
C12	0.6556 (3)	0.40662 (7)	0.87927 (14)	0.0447 (4)	
H12A	0.5258	0.3966	0.8551	0.054*	
H12B	0.7339	0.3740	0.8676	0.054*	
C13	0.7202 (2)	0.47953 (6)	1.00090 (11)	0.0285 (3)	
C14	0.73685 (19)	0.51947 (5)	0.50739 (11)	0.0223 (3)	
H14	0.7641	0.5005	0.5737	0.027*	
C15	0.75223 (18)	0.48975 (5)	0.41639 (10)	0.0209 (3)	
H15	0.7254	0.5094	0.3507	0.025*	
C16	0.80663 (17)	0.43002 (5)	0.41021 (10)	0.0192 (2)	
C17	0.82357 (18)	0.40565 (5)	0.30881 (10)	0.0215 (3)	
H17	0.7998	0.4283	0.2466	0.026*	
C18	0.87439 (19)	0.34909 (5)	0.29830 (10)	0.0232 (3)	
H18	0.8852	0.3345	0.2296	0.028*	
C19	0.91014 (18)	0.31313 (5)	0.39002 (10)	0.0210 (3)	
C20	0.89460 (19)	0.33767 (5)	0.49262 (10)	0.0232 (3)	
H20	0.9185	0.3152	0.5550	0.028*	
C21	0.84471 (19)	0.39423 (5)	0.50171 (10)	0.0230 (3)	
H21	0.8360	0.4092	0.5704	0.028*	
C22	0.9988 (2)	0.23508 (6)	0.27733 (13)	0.0367 (4)	
H22A	0.8892	0.2382	0.2238	0.055*	
H22B	1.0363	0.1956	0.2849	0.055*	
H22C	1.0985	0.2573	0.2546	0.055*	
C23	1.0080 (2)	0.22143 (6)	0.47718 (13)	0.0299 (3)	
H23A	0.9014	0.2173	0.5150	0.045*	
H23B	1.1081	0.2396	0.5241	0.045*	
H23C	1.0480	0.1843	0.4562	0.045*	

### Atomic displacement parameters (Å^2^)

**Table:**

	*U*^11^	*U*^22^	*U*^33^	*U*^12^	*U*^13^	*U*^23^
O1	0.0397 (6)	0.0184 (4)	0.0163 (4)	0.0043 (4)	0.0042 (4)	0.0032 (3)
O2	0.0657 (8)	0.0242 (5)	0.0325 (6)	−0.0058 (5)	0.0006 (6)	0.0083 (4)
O3	0.0744 (9)	0.0343 (6)	0.0218 (5)	−0.0004 (6)	0.0059 (6)	0.0032 (4)
O1W	0.0989 (12)	0.0353 (7)	0.0591 (9)	−0.0211 (7)	0.0422 (9)	−0.0125 (6)
N1	0.0253 (6)	0.0156 (5)	0.0198 (5)	0.0005 (4)	0.0044 (4)	−0.0002 (4)
N2	0.0262 (6)	0.0169 (5)	0.0173 (5)	0.0008 (4)	0.0041 (4)	0.0008 (4)
N3	0.0408 (7)	0.0209 (5)	0.0192 (5)	−0.0012 (5)	0.0027 (5)	0.0024 (4)
N4	0.0439 (7)	0.0178 (5)	0.0278 (6)	0.0079 (5)	0.0076 (6)	−0.0010 (4)
C1	0.0207 (6)	0.0158 (5)	0.0172 (5)	−0.0004 (4)	0.0026 (5)	−0.0005 (4)
C2	0.0317 (7)	0.0200 (6)	0.0162 (6)	0.0013 (5)	0.0037 (5)	0.0000 (4)
C3	0.0321 (7)	0.0201 (6)	0.0188 (6)	0.0023 (5)	0.0026 (5)	0.0042 (4)
C4	0.0294 (7)	0.0159 (5)	0.0238 (6)	0.0031 (5)	0.0051 (5)	0.0008 (4)
C5	0.0282 (7)	0.0186 (5)	0.0189 (6)	0.0027 (5)	0.0058 (5)	−0.0015 (4)
C6	0.0197 (6)	0.0167 (5)	0.0170 (6)	−0.0001 (4)	0.0026 (5)	0.0013 (4)
C7	0.0214 (6)	0.0158 (5)	0.0195 (6)	−0.0007 (4)	0.0035 (5)	−0.0005 (4)
C8	0.0229 (6)	0.0181 (5)	0.0175 (6)	−0.0003 (4)	0.0034 (5)	0.0020 (4)
C9	0.0417 (8)	0.0205 (6)	0.0173 (6)	0.0018 (5)	0.0044 (6)	0.0012 (4)
C10	0.0359 (8)	0.0261 (7)	0.0229 (7)	−0.0056 (6)	−0.0006 (6)	0.0047 (5)
C11	0.0535 (10)	0.0285 (7)	0.0251 (7)	−0.0012 (7)	0.0024 (7)	−0.0029 (5)
C12	0.0713 (13)	0.0238 (7)	0.0367 (9)	0.0001 (7)	−0.0008 (9)	−0.0008 (6)
C13	0.0366 (8)	0.0239 (6)	0.0248 (7)	0.0036 (6)	0.0038 (6)	0.0067 (5)
C14	0.0281 (7)	0.0154 (5)	0.0235 (6)	0.0019 (5)	0.0044 (5)	0.0018 (4)
C15	0.0231 (6)	0.0168 (5)	0.0229 (6)	0.0000 (5)	0.0031 (5)	0.0017 (4)
C16	0.0212 (6)	0.0156 (5)	0.0209 (6)	−0.0009 (4)	0.0034 (5)	−0.0006 (4)
C17	0.0262 (7)	0.0203 (6)	0.0181 (6)	0.0003 (5)	0.0038 (5)	0.0018 (4)
C18	0.0280 (7)	0.0225 (6)	0.0196 (6)	0.0017 (5)	0.0050 (5)	−0.0022 (4)
C19	0.0230 (6)	0.0166 (5)	0.0236 (6)	0.0003 (5)	0.0040 (5)	−0.0019 (4)
C20	0.0317 (7)	0.0186 (6)	0.0193 (6)	0.0010 (5)	0.0036 (5)	0.0020 (4)
C21	0.0314 (7)	0.0194 (6)	0.0185 (6)	−0.0001 (5)	0.0048 (5)	−0.0019 (4)
C22	0.0492 (10)	0.0269 (7)	0.0354 (8)	0.0130 (6)	0.0114 (7)	−0.0064 (6)
C23	0.0334 (8)	0.0194 (6)	0.0368 (8)	0.0039 (5)	0.0048 (6)	0.0037 (5)

### Geometric parameters (Å, °)

**Table:**

O1—C8	1.3512 (14)	C9—H9A	0.9700
O1—C9	1.4428 (15)	C9—H9B	0.9700
O2—C13	1.3532 (17)	C10—H10A	0.9700
O2—C12	1.443 (2)	C10—H10B	0.9700
O3—C13	1.2130 (17)	C11—C12	1.522 (2)
O1W—H11	0.867 (10)	C11—H11A	0.9700
O1W—H12	0.861 (10)	C11—H11B	0.9700
N1—C7	1.3158 (15)	C12—H12A	0.9700
N1—C1	1.3754 (15)	C12—H12B	0.9700
N2—C8	1.2922 (15)	C14—C15	1.3422 (17)
N2—C6	1.3757 (15)	C14—H14	0.9300
N3—C13	1.3401 (16)	C15—C16	1.4523 (16)
N3—C11	1.4507 (18)	C15—H15	0.9300
N3—C10	1.4522 (17)	C16—C17	1.4008 (16)
N4—C19	1.3760 (16)	C16—C21	1.4042 (17)
N4—C22	1.4447 (17)	C17—C18	1.3803 (17)
N4—C23	1.4514 (18)	C17—H17	0.9300
C1—C2	1.4083 (16)	C18—C19	1.4074 (17)
C1—C6	1.4170 (15)	C18—H18	0.9300
C2—C3	1.3734 (17)	C19—C20	1.4138 (16)
C2—H2	0.9300	C20—C21	1.3762 (17)
C3—C4	1.4082 (17)	C20—H20	0.9300
C3—H3	0.9300	C21—H21	0.9300
C4—C5	1.3739 (17)	C22—H22A	0.9600
C4—H4	0.9300	C22—H22B	0.9600
C5—C6	1.4068 (16)	C22—H22C	0.9600
C5—H5	0.9300	C23—H23A	0.9600
C7—C14	1.4536 (16)	C23—H23B	0.9600
C7—C8	1.4575 (16)	C23—H23C	0.9600
C9—C10	1.504 (2)		
			
C8—O1—C9	117.20 (9)	C12—C11—H11A	111.5
C13—O2—C12	109.03 (11)	N3—C11—H11B	111.5
H11—O1W—H12	108 (2)	C12—C11—H11B	111.5
C7—N1—C1	118.03 (10)	H11A—C11—H11B	109.3
C8—N2—C6	116.22 (10)	O2—C12—C11	105.82 (12)
C13—N3—C11	112.01 (11)	O2—C12—H12A	110.6
C13—N3—C10	121.85 (12)	C11—C12—H12A	110.6
C11—N3—C10	124.02 (11)	O2—C12—H12B	110.6
C19—N4—C22	119.33 (11)	C11—C12—H12B	110.6
C19—N4—C23	120.48 (11)	H12A—C12—H12B	108.7
C22—N4—C23	118.58 (11)	O3—C13—N3	128.51 (13)
N1—C1—C2	119.81 (10)	O3—C13—O2	120.80 (12)
N1—C1—C6	121.16 (10)	N3—C13—O2	110.69 (12)
C2—C1—C6	119.03 (10)	C15—C14—C7	124.57 (12)
C3—C2—C1	120.75 (11)	C15—C14—H14	117.7
C3—C2—H2	119.6	C7—C14—H14	117.7
C1—C2—H2	119.6	C14—C15—C16	126.51 (12)
C2—C3—C4	119.93 (11)	C14—C15—H15	116.7
C2—C3—H3	120.0	C16—C15—H15	116.7
C4—C3—H3	120.0	C17—C16—C21	116.87 (11)
C5—C4—C3	120.56 (11)	C17—C16—C15	119.68 (11)
C5—C4—H4	119.7	C21—C16—C15	123.45 (11)
C3—C4—H4	119.7	C18—C17—C16	122.05 (11)
C4—C5—C6	120.27 (11)	C18—C17—H17	119.0
C4—C5—H5	119.9	C16—C17—H17	119.0
C6—C5—H5	119.9	C17—C18—C19	120.94 (11)
N2—C6—C5	119.74 (10)	C17—C18—H18	119.5
N2—C6—C1	120.82 (10)	C19—C18—H18	119.5
C5—C6—C1	119.44 (11)	N4—C19—C18	121.65 (11)
N1—C7—C14	121.53 (11)	N4—C19—C20	121.16 (11)
N1—C7—C8	119.17 (10)	C18—C19—C20	117.19 (11)
C14—C7—C8	119.30 (11)	C21—C20—C19	121.11 (11)
N2—C8—O1	120.66 (10)	C21—C20—H20	119.4
N2—C8—C7	124.58 (11)	C19—C20—H20	119.4
O1—C8—C7	114.76 (10)	C20—C21—C16	121.83 (11)
O1—C9—C10	107.50 (11)	C20—C21—H21	119.1
O1—C9—H9A	110.2	C16—C21—H21	119.1
C10—C9—H9A	110.2	N4—C22—H22A	109.5
O1—C9—H9B	110.2	N4—C22—H22B	109.5
C10—C9—H9B	110.2	H22A—C22—H22B	109.5
H9A—C9—H9B	108.5	N4—C22—H22C	109.5
N3—C10—C9	114.60 (12)	H22A—C22—H22C	109.5
N3—C10—H10A	108.6	H22B—C22—H22C	109.5
C9—C10—H10A	108.6	N4—C23—H23A	109.5
N3—C10—H10B	108.6	N4—C23—H23B	109.5
C9—C10—H10B	108.6	H23A—C23—H23B	109.5
H10A—C10—H10B	107.6	N4—C23—H23C	109.5
N3—C11—C12	101.37 (12)	H23A—C23—H23C	109.5
N3—C11—H11A	111.5	H23B—C23—H23C	109.5
			
C7—N1—C1—C2	179.80 (12)	C13—N3—C11—C12	9.09 (18)
C7—N1—C1—C6	−0.09 (18)	C10—N3—C11—C12	172.75 (14)
N1—C1—C2—C3	179.12 (12)	C13—O2—C12—C11	8.34 (19)
C6—C1—C2—C3	−0.99 (19)	N3—C11—C12—O2	−10.11 (18)
C1—C2—C3—C4	0.1 (2)	C11—N3—C13—O3	175.00 (16)
C2—C3—C4—C5	0.4 (2)	C10—N3—C13—O3	10.9 (3)
C3—C4—C5—C6	−0.1 (2)	C11—N3—C13—O2	−4.46 (18)
C8—N2—C6—C5	179.13 (12)	C10—N3—C13—O2	−168.53 (13)
C8—N2—C6—C1	−0.61 (18)	C12—O2—C13—O3	177.73 (16)
C4—C5—C6—N2	179.43 (12)	C12—O2—C13—N3	−2.76 (19)
C4—C5—C6—C1	−0.83 (19)	N1—C7—C14—C15	−4.4 (2)
N1—C1—C6—N2	0.96 (18)	C8—C7—C14—C15	175.96 (13)
C2—C1—C6—N2	−178.93 (11)	C7—C14—C15—C16	−179.58 (12)
N1—C1—C6—C5	−178.77 (12)	C14—C15—C16—C17	−177.43 (13)
C2—C1—C6—C5	1.34 (18)	C14—C15—C16—C21	2.4 (2)
C1—N1—C7—C14	179.37 (11)	C21—C16—C17—C18	0.28 (19)
C1—N1—C7—C8	−0.99 (18)	C15—C16—C17—C18	−179.85 (12)
C6—N2—C8—O1	178.87 (11)	C16—C17—C18—C19	0.5 (2)
C6—N2—C8—C7	−0.53 (19)	C22—N4—C19—C18	9.7 (2)
C9—O1—C8—N2	−2.79 (18)	C23—N4—C19—C18	174.98 (12)
C9—O1—C8—C7	176.67 (11)	C22—N4—C19—C20	−170.81 (14)
N1—C7—C8—N2	1.4 (2)	C23—N4—C19—C20	−5.5 (2)
C14—C7—C8—N2	−178.95 (12)	C17—C18—C19—N4	178.58 (13)
N1—C7—C8—O1	−178.04 (11)	C17—C18—C19—C20	−0.97 (19)
C14—C7—C8—O1	1.61 (17)	N4—C19—C20—C21	−178.92 (13)
C8—O1—C9—C10	−157.88 (11)	C18—C19—C20—C21	0.64 (19)
C13—N3—C10—C9	−133.37 (14)	C19—C20—C21—C16	0.2 (2)
C11—N3—C10—C9	64.51 (19)	C17—C16—C21—C20	−0.62 (19)
O1—C9—C10—N3	−67.73 (15)	C15—C16—C21—C20	179.52 (12)

### Hydrogen-bond geometry (Å, °)

**Table:**

*D*—H···*A*	*D*—H	H···*A*	*D*···*A*	*D*—H···*A*
O1w—H11···N1	0.87 (1)	2.18 (1)	3.039 (2)	170 (2)
O1w—H12···O3^i^	0.86 (1)	1.96 (1)	2.820 (2)	176 (3)

Symmetry codes: (i) *x*, *y*, *z*−1.
